# Comparison of the Efficacy of the Various Treatment Modalities in the Management of Perianal Crohn’s Fistula: A Review

**DOI:** 10.7759/cureus.11882

**Published:** 2020-12-03

**Authors:** Shah Huzaifa Feroz, Asma Ahmed, Abilash Muralidharan, Pragatheeshwar Thirunavukarasu

**Affiliations:** 1 General Surgery, Jawaharlal Nehru Medical College, Aligarh, IND; 2 General Surgery, Larkin Community Hospital, Miami, USA; 3 General Surgery, Ramaiah Medical College and Hospital, Bangalore, IND; 4 Internal Medicine, Kiruba Hospital, Coimbatore, IND; 5 Surgical Oncology, Cancer Treatment Centers of America, Tulsa, USA

**Keywords:** perianal fistula, crohn's disease, seton, biologicals, infliximab, vedolizumab, fistulotomy, ustekinumab

## Abstract

Crohn's disease (CD) is a transmural inflammatory bowel disease (IBD) that can affect any part of the gastrointestinal (GI) tract. With the disease's progression, adhesions and transmural fissuring, intra-abdominal abscesses, and fistula tracts may develop. An anal fistula (or fistula-in-ano) is a chronic abnormal epithelial lined tract communicating the anorectal lumen (internal opening) to the perineal or buttock skin (external opening). The risk of fistula development varies from 14%-38%. It can cause significant morbidity, which adversely impacts the quality of life. It is mostly believed that an anal crypt gland infection causes anal abscesses, leading to fistula development. Crohn's disease's pathogenesis involves Th1 and Th17 hypersensitivity due to an unknown antigen within the intestinal mucosa.

Evidence to support this review was gathered via the Pubmed database. Search terms used were combinations of "Perianal fistula," "seton," "immunotherapy." Studies were reviewed and cross‐referenced for additional reports.

Setons are surgical thread loops passed from the external to the internal opening of the fistula tract and exteriorized through the anorectal canal, facilitating abscess drainage and inciting a local inflammatory reaction, thus promoting the resolution of the fistula. Biologicals such as anti-tumor necrosis factor (TNF) antibody (infliximab, adalimumab, certolizumab), anti-IL-12/23 (ustekinumab), and anti-α₄β₇ integrin antibody (vedolizumab) have been approved for Crohn's disease targeting the Th1/Th17-mediated inflammation. Other therapeutic modalities are fistulotomy, cyanoacrylate glue, bioprosthetic plugs, mucosal advancement flap, ligation of inter-sphincteric fistula tract (LIFT), diverting stoma, proctectomy, video-assisted anal fistula treatment (VAAFT), and fistula laser closure (FiLaC).

Our review found that chronic seton therapy should be the primary approach, especially if the patient has a perianal abscess. It has a low incidence of re-intervention, recurrent abscess formation, and side-branching of the fistulous tract, with preservation of the fistulous tract's patency and cost-effectiveness. The major disadvantage of seton therapy is the discomfort and time to achieve stability. Among the biologicals, infliximab is the only therapy which has a statistically significant effect on the healing rate of perianal Crohn's fistula compared to placebo, but the major disadvantage associated with anti-TNF as sole therapy is high re-intervention rate, prolong maintenance therapy, high recurrence rate, and severe side effects. We hypothesize that the two aspects should be addressed concurrently to increase the fistula healing or closure rate. First, the seton should be used as initial therapy to maintain tract patency to allow abscess drainage and minimize the intestinal flora colonization within the tract mucosa, thereby leukocytic infiltration and propagation of inflammation within the tract. The second aspect that has to be considered is that we should target the initial stimulation of the Th1/Th17 mediated hypersensitivity instead of a factor/cytokine involved in the inflammation mediation. Although the unknown antigen triggering such hypersensitivity is not clear, we could target the RAR-related orphan receptor γ (RORγ)-T (transcription factor involved in activation of Th17 cells) and the T-bet (transcription factor involved in activation of Th17 cells) within the GI mucosa by a novel target immune therapy.

## Introduction and background

The doctrine of fistula-in-ano treatment is to close the fistula tract without compromising continence [1]. The data from the National Health Interview Survey (NHIS) in 2015 revealed an estimated 3.1 million, or 1.3%, of US adults have inflammatory bowel disease (IBD), with a significant increase in prevalence and hospitalization rate from 1999 [2,3,4]. The prevalence also differed significantly among several sociodemographic factors [2]. The mean hospitalization costs were $11,345 for Crohn's disease (CD). It increased annually by 3% from 2003 to 2008, although unchanged between 2008 to 2014 [5].

In 2017, it was estimated that there were ~76,600 prevalent cases and ~15,700 incident cases of fistulizing CD in the US, with varying distribution according to the fistula type. ~11.7% of US individuals with CD have fistulizing CD at a given time (8.1% anal, 1.1% rectovaginal, 0.3% enterocutaneous, and 2.2% internal of the Crohn's population) [6]. After one year of diagnosis, the cumulative incidence of fistulas was 21%, while 50% after 20 years of diagnosis [6]. It is estimated that ~75% of anal fistulas in the CD population are complex [7,8].

**Figure 1:**
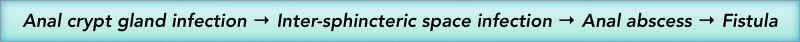
The sequence of events leading to perianal fistula formation

CD is a transmural IBD that can affect any part of the gastrointestinal (GI) tract­ from the mouth to the anus, but most commonly involves terminal ileum. It exhibits bimodal distribution first peak between the second and third decade and the second peak between the sixth and seventh decade. Three distinct patterns of disease are seen, i.e., inflammatory, stricturing, and perforating. With the disease's progression, adhesions and transmural fissuring, intra-abdominal abscesses, and fistula tracts may develop. An anal fistula (or fistula-in-ano) is a chronic abnormal epithelial lined tract (may also have granulation tissue) communicating the anorectal lumen (internal opening) to the perineal or buttock skin (external opening) and rarely to the vagina (in women) [9]. The lifetime risk of fistula development in patients with CD ranges from 14% to 38% [10]. The perianal fistulizing Crohn's disease can cause pain, purulent discharge, and destruction of the sphincter and perineal tissue, resulting in a significant adverse effect on the quality of life [11], and is a predictor of poor long-term outcomes [12]. It is mostly believed that anal abscess caused by an anal crypt gland infection leads to the suppuration into the inter-sphincteric space, forming an abscess, leading to fistula development (Figure 1) [13].

The pathogenesis of Crohn's disease involves Th1 and Th17 hypersensitivity due to an unknown antigen (possibly enteric floral antigens) within the intestinal mucosa. Increased production of transforming growth factor-β (TGF-β) and Interleukin-6 (IL-6) is responsible for the commitment of naive T-helper cells (Th0 cells) to Th17 cells, while IL-12 is required for differentiation of a Th0 cell into a Th1 cell. The production of IL-21 and IL-23 is responsible for the maintenance and expansion of the Th17 cells, while tumor necrosis factor (TNF) mediates the inflammation [14]. In the inflammatory infiltrate, IL-12, TNF, and IL‑13 induce epithelial-to-mesenchymal transition and upregulation of matrix metalloproteinases, leading to tissue remodeling and fistula formation [15].

Classifications of perianal fistula

**Figure 2:**
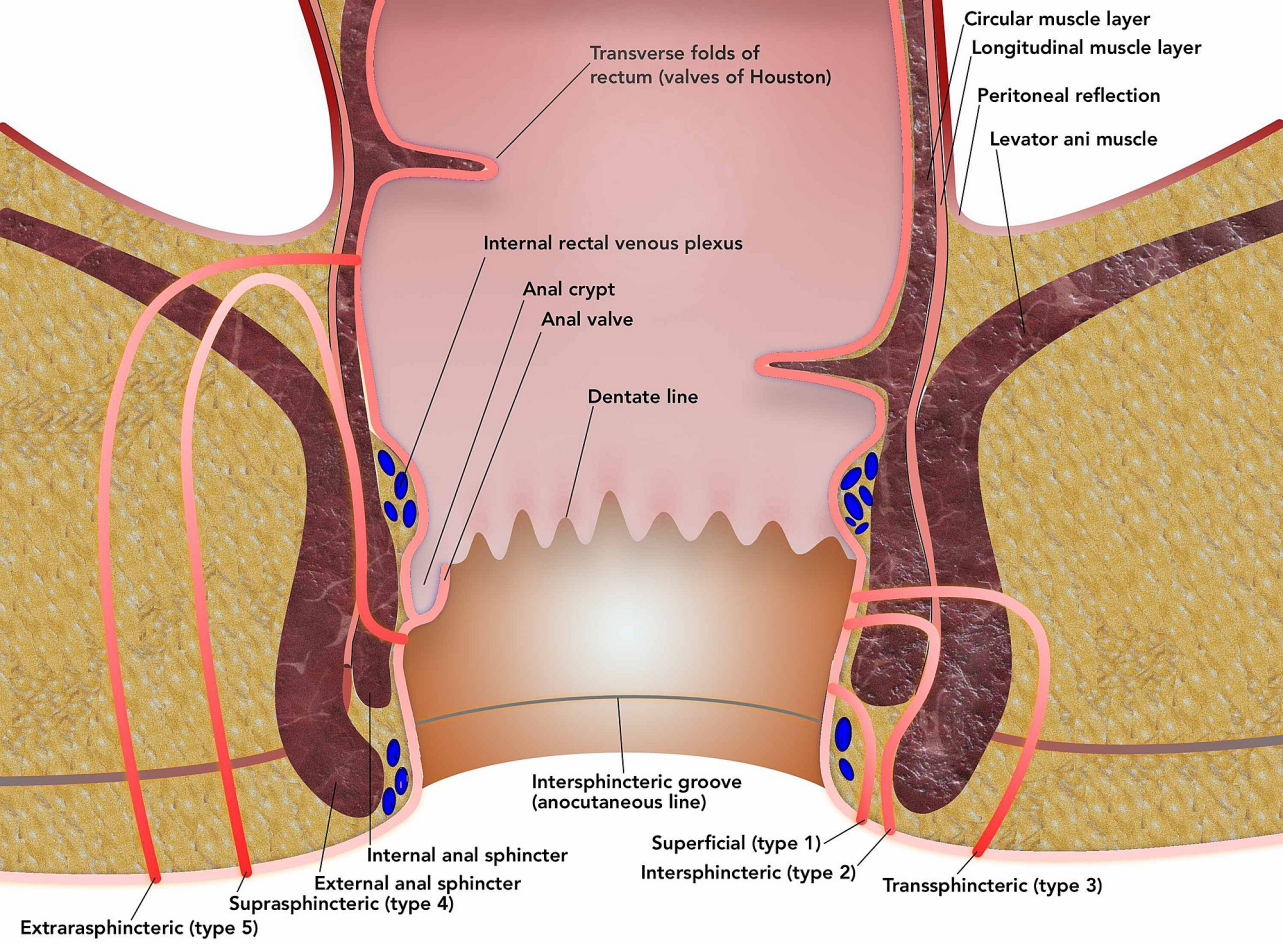
Park's Classification

Park's fistula classification [16] is based on the fistula's relation to the external sphincter muscle and is a more anatomically precise classification (Table 1, Figure 2). The American Gastroenterological Association (AGA) Classification [17] for perianal fistulas is based on the complexity (Table 2), and is widely used as an empirical classification and includes a physical examination of the perianal area. Classification of the fistula based on four types, based on the relation of the fistula to the sphincter muscle; aka Park's Classification)

**Table 1:** Park's Classification

Park’s Classification		
Type	Name	Comments
I	Superficial	Fistula tract does not traverses through any sphincter or musculature
II	Intersphincteric (most common)	Fistula tract traverses between the internal and external anal sphincter through the intersphincteric plane
III	Transsphincteric	Fistula tract traverses through the external anal sphincter
IV	Suprasphincteric	Fistula starts in the intersphincteric plane and then passes upward to a point above the puborectalis muscle, and then laterally over this muscle and downward between the puborectal and levator muscles into the ischiorectal fossa
V	Extrasphincteric	Fistula passes from the perineal skin through the ischio­rectal fossa and levator ani muscle, and finally penetrates the rectal wall. May arise from trauma, foreign body, pelvic abscess, or cryptoglandular abscess

**Table 2:** American Gastroenterological Association’s (AGA) Classification [17]

American Gastroenterological Association’s (AGA) Classification		
Simple		Complex
Low (superficial or low intersphincteric or low transsphincteric origin of the fistula tract) with:	Single external opening	High (high intersphincteric or high transsphincteric or extrasphincteric or suprasphincteric origin of the fistula tract)
	No pain or fluctuation to suggest perianal abscess	May have multiple external openings
	No evidence of a rectovaginal fistula	May be associated with the presence of pain or fluctuation to suggest a perianal abscess
	No evidence of anorectal stricture.	May be associated with the presence of a rectovaginal fistula
	The presence of active rectal Crohn’s disease may make a simple fistula more complicated to manage.	May be associated with the presence of an anorectal stricture
		May be associated with the presence of active rectal disease at endoscopy

Setons are surgical thread loops passed from the external to the internal opening of the fistula tract and exteriorized through the anorectal canal, facilitating abscess drainage and inciting a local inflammatory reaction [18], thus promoting the resolution of the fistula. Surgeons usually prefer setons when the patient has a concomitant perianal abscess since it also allows incision and drainage. In addition, antibiotics (metronidazole or ciprofloxacin) are also supplemented, which further promotes healing [15].

The biologicals, e.g., anti-TNF antibody (infliximab, adalimumab, certolizumab), anti-IL-12/23 (ustekinumab), and anti-α₄β₇ integrin antibody (vedolizumab) have been approved for Crohn's disease targeting the Th1/Th17 mediated inflammation and the diapedesis of the leukocytes in the intestinal mucosa respectively. With superficial and low inter-sphincteric fistulas, fistulotomy is often the choice, especially in the absence of active proctitis and a failed antibiotic therapy or any local therapy. Other modalities to manage the perianal Crohn's fistula are cyanoacrylate glue, bioprosthetic plugs, mucosal advancement flap, ligation of inter-sphincteric fistula tract (LIFT), diverting stoma, proctectomy, video-assisted anal fistula treatment (VAAFT), and fistula laser closure (FiLaC) [15].

## Review

Methods

Evidence to support this review was collected via the Pubmed database. Search terms used were combinations of "Perianal fistula," "seton," "immunotherapy." Studies discussing the different modalities of management underpinning perianal fistula, especially in Crohn's disease, were reviewed and cross‐referenced for additional reports. Table 3 depicts the keywords used and the number of articles found on the PubMed database with a filter of the 20-year publication date and human studies. All the data were collected after a meticulous review of the articles.

**Table 3:** Number of articles found on the PubMed Database related to our Keywords

Keyword	Database	Number of articles
Perianal fistula	PubMed	1673
Perianal fistua seton	PubMed	178
Perianal fistua seton biologicals	PubMed	33

Discussion

Seton Placement

Setons have been considered the mainstay of surgical management for most fistulae-in-ano. There are mainly two techniques for seton placement based on the knot's tightness and the cutting nature. The first is the cutting or tight seton technique, in which a non-absorbable thread is inserted into the fistula tract and exteriorized through the anorectal canal, which incites inflammation and fibrosis. The setons are subsequently tightened, causing gradual, controlled cutting of the sphincter (staged fistulotomy) while allowing the reactive fibrosis to occur, ensuring the sphincter integrity [19].

In a study done by Raslan et al. with 28 patients with complex perianal Crohn's fistula, a 90.2% healing rate was noted by the end of the study (one year), with a recurrence rate of 9.8%. A direct correlation between the healing time and the distance from the anal verge was also observed [13]. There have been many studies indicating complications associated with this technique, including prolonged perianal pain and incontinence. Although the rate of the incontinence associated varied, some reported minor damage 15.7% to flatus, 5.9% to liquid stools, and no incontinence to solid stool [13]; others indicated a significant incontinence rate of 20-67% [20] and 58% (103/178) [21] of varying degrees.

The second technique is the loose seton technique developed to avoid cutting the anal sphincter, thereby theoretically reducing incontinence risk. It can be either placed as a long-term indwelling seton [22] or used as a two-staged fistulotomy [23]. Indwelling seton inhibits the pus collection and promotes continuous drainage of the abscess, hence usually placed after the abscess drainage. In some cases, it results in closure of the fistula, generally within six to 12 weeks [24]. In a study by Thornton et al., 28 cases of complex perianal Crohn's fistula had long term indwelling setons, and 92% cases (26/28) reported an improvement in symptoms at a median follow-up of 13 months [22], while in another study by Fazio et al., fistula closure was reported in 8% (16/194) cases [25]. In a case series led by Kelly et al., 7% (14/200) had spontaneous resolution of the fistula tract with seton placement alone [1]. The study by Papaconstantinou et al. reported improvement in all 25 cases of mid- or low-level trans-sphincteric fistula in CD, with recurrence six months after seton removal in one patient, and minor incontinence was found in 12% (3/25) [26]. Incontinence rates associated with loose setons have been significantly lower, varied from 5-17%, compared to cutting setons [27,28]. No patient reported a deterioration in fecal continence after seton insertion in a study by Thornton et al. and Kelly et al.

The two-stage seton fistulotomy is more commonly used. Besides continuous drainage, the loose seton placement also incites a fibrotic reaction, which may lead to primary closure or promote migration of the fistula tract superficially, usually within six to eight weeks, after which fistulotomy or fistulectomy can be safely performed. In a study by Kelly et al., 93% (186/200) underwent two-staged seton fistulotomy, which results in the clearance of fistula in all patients. Only 4% (eight) described minor urgency, and none reported incontinence at follow-up [1]. Although the study conducted by Galis-Rozen et al., in the Crohn's group, two-staged seton fistulotomy, only 59% (10) showed significant clinical improvement while 35% (six) showed no improvement [24].

Recently, many surgeons have stopped using the cutting seton technique due to their association with postoperative discomfort and the increased risk of incontinence. Still, many surgeons prefer to use this technique. Incontinence rates associated with loose setons have been significantly lower (5-17%) compared to cutting setons (20-67%) [27,28]. It is important to note that most studies conducted before 2000 agree that cutting seton is at a more disadvantageous position than loose setons, but no convincing evidence of superior efficacy [29] or reduced sphincter destruction has been found in later subsequent studies. It is supported by the fact that the studies showed no statistical significance or consisted of a small sample size (low power) to confer strong statistical relevance. In a case series of 59 patients, Drager et al. reported no significant difference in function or healing (92%) or recurrence rates between the two methods [30]. However, the loose setons' placement remained the preferred choice of surgeons, and many consider it the gold standard for complex fistula [31], even when used as a combination of medical and surgical treatment. In a broader context, loose seton placement, especially as a two-staged seton fistulotomy for a complex fistula, is cost-efficient, well-tolerated, and efficacious (Table 4).

**Table 4:** Clinical studies on the management of perianal Crohn's fistula primarily by the Seton's placement or the combination of therapies ¥ Published date; CD (Crohn’s Disease); non-CD (Non-Crohn’s Disease); PDAI (Perianal Disease Activity Index), TNF (tumor necrosis factor)

Studies	Method¥	Patient Characteristics	Key Findings with Complications (if any)	Limitations
Thornton et al. [22]	Case series (2005)	28 patients with 43 complex perianal Crohn’s fistulas (5 intersphincteric, 10 transsphincteric, 19 suprasphincteric, 5 extrasphincteric, and 4 rectovaginal fistulas) Median age: 36 (range, 18–72) years.	Managed with long-term indwelling setons or mushroom catheters.	9 patients of perianal Crohn's disease were also treated perioperatively with adjuvant therapy, while only 1 patient was treated with both infliximab and interleukin-2. A total of 5 patients were receiving prednisone and or Imuran at the time of the surgery. Metronidazole was used perioperatively in 9 patients, and 2 patients were concomitantly treated with ciprofloxacin.
			92% of cases (26) reported alleviation in their perianal symptoms at a median follow-up of 13 months (2–81), as determined by clinicians' qualitative evaluation. At the time of diagnosis, the median anal wall thickness was 18.5 mm, reduced to 14 mm after seton insertion, and symptom control (P < 0.02). 21% of cases (6) developed recurrent or new perianal symptoms, while the seton was in situ. 11% of patients (3) required further surgical intervention.	
			No patient reported a deterioration in fecal continence after seton insertion.	
			Patient age (P < 0.005), reduction in anal wall thickness after seton insertion (P < 0.04), and length of follow-up (P < 0.03) were notable predictors of long term symptom control, in a multivariate analysis.	
			For complex perianal Crohn’s fistulas, long-term indwelling seton is an effective management modality, which does not negatively impact fecal continence.	
Van Der Hagen et al. [23]	Case series (2005)	30 patients with a complex perianal fistulas. (median age; 42 years, range 22–68 years). 7 had Crohn’s disease without signs of rectal and anal involvement other than the fistula.	Two-staged procedure; the initial treatment consisted of a non-cutting seton with or without a diverting stoma, followed by (5 months later) definitive surgical treatment consisted of an advancement flap (26) or fistulotomy (4).	
			In 97% of cases (29/30), the wounds had healed completely within 3 months at a median follow up of 22 months (8−52); 23% of cases (7/30) subsequently developed a recurrent fistula (2 of 7 was CD patient) and minor soiling occurred in 23% (7/30) patients.	
Galis-Rozen et al. [24]	Case series with comparison (2009)	77 patients with a complex fistula out of which 60 (42 males) were in the non-CD group and 17 (12 males) were in the CD group.	Analysis of the results of permanent loose seton in the management of high anal fistulas in CD patients and two-stage seton fistulotomy in patients without CD. CD group: 17 underwent 29 fistula-related procedures (1−4 procedure/ patient with a median of 1). 59% (10) showed significant clinical improvement, 35% (6) showed no improvement, while in 6% (1), the condition worsened. During the follow-up, 40% (7) cases had additional procedures.	
			During a median follow-up of 24 months (6–48), 14/77 (18%) patients (9 non-CD, 5 CD) experienced long-term morbidity. Among the CD patients, 5 developed a perianal abscess, which required surgical drainage in 4 of which 1 developed fecal incontinence. While among non-CD, 5 required perianal abscess drainage, and another 4 developed minor fecal incontinence.	
			The approach to complex perianal fistula in CD should be primarily conservative to induce remission and resolution of the fistula. At the same time, in non-CD, it should be surgical. With these tactics, surgery becomes an option when drug therapy fails.	
Chung et al. [32]	Retrospective cohort (2010)	51 patients of IBD with anal fistulas were identified, and compared with a control group of 232 patients with non-IBD perianal fistula. The median age was 39 years (range 21-66)	At 12 weeks in the treatment group for the seton drain (40 cases), flap advancement (5 cases), fistula plug (4 cases), and fibrin glue (2 cases), the postoperative healing rates were 28%, 20%, 75%, and 0%, respectively. These procedures did not alter continence scores.	Healing rates were not statistically significantly different between the 4 treatment groups. Small sample size and low power to confer strong statistical relevance Disproportionate distribution of patients among groups.
Kelly et al. [1]	Case series (2014) at multicenters	200 patients with anal fistula, out of which 46 patients (23%) were of CD. 69.5 % (n = 139) were males, and mean age was 42.6 years. 85 (42.5 %) were intersphincteric, 71 (35.5 %) were transphincteric, 16 (8 %) were extrasphincteric, and 12 (6 %) were suprasphinc- teric. In 16 patients (8 %), the location of the fistula tract was not documented.	Managed with loose seton placement. 96% of patients (187/200) tolerated the procedure with no complications, while 3% (6/200) had a local reaction secondary to the seton material requiring it to be changed, and 1 % (2/200) could not tolerate the presence of seton due to significant perianal discomfort.	Lack of long-term follow-up Retrospective
			All patients had a successful clearance of fistula. 93% (186/200) had a controlled fistulotomy when there was minimal sphincter involvement, while the remaining 7% (14/200) had spontaneous resolution of the fistula tract with seton placement alone.	
			At a 6-monthly follow-up, the fistula recurrence rate was noted to be 6% (12), while only 4% (8) reported minor urgency, and none reported incontinence at follow-up.	
Raslan et al. [13]	Case series (2016)	51 patients with high perianal fistula Conducted over 12 months	Managed with cutting seton insertion technique. 90.2% healing rate (complete cure) by the end of the study, with a recurrence rate of 9.8%. A direct correlation was noticed between the healing time and the distance from the anal verge.	Patients with low perianal fistula were excluded Crohn’s disease patient were excluded.
			In postoperative patients, the incontinence was 15.7% to flatus, 5.9% to liquid stools with no incontinence to solid stools.	
Papaconstantinou et al. [26]	Case series (2016)	59 patients of perianal Crohn’s fistula were identified. High transsphincteric fistula (44%), mid or low transsphincteric fistulas (51%) and rectovaginal fistula (5%).	Managed with loose seton placement (all cases of high trans-sphincteric fistulas), fistulotomy, or seton placement based on the clinical evaluation (all cases with mid- or low-level trans-sphincteric fistula).	All patients included in this study were administered ciprofloxacin 400 mg BD and metronidazole 500 mg TDS, with the first dose being given preoperatively and continued postoperatively for at least 7 days.
			Out of 59 cases, 29, 1, 25, and 4 underwent fistulotomy, fistulotomy with a proximal diverting colostomy, seton placement, and seton placement with a proximal diverting colostomy, respectively.	
			Seton placement is more appropriate and is the only option for more high-lying or complicated fistulae. Fistulotomy could achieve good outcomes in wound healing and incontinence in strictly selected patients with CD suffering from low-lying trans-sphincteric fistulae.	
			The mean follow-up duration was 1.6 ± 1.1 years. One patient in the seton placement group experienced recurrence six months after seton removal, and one patient in the fistulotomy group failed to achieve wound healing. Minor incontinence was noticed in six patients treated with fistulotomy and in three patients treated with seton placement; however, this difference was not significant (chi-square = 1.723, p = 0.273).	
Wasmann et al. [10]	Randomized controlled trial (2020)	44 of the 126 planned patients with high perianal Crohn’s fistulas with a single internal opening were randomly assigned between 2013-2017. 50 of the 126 declined randomization due to treatment preference hence were included in a parallel prospective PISA registry cohort.	Before randomization, all patients underwent seton insertion [vessel loop] under general anesthesia in a daycare setting and received a 2-week antibiotic course. Furthermore, 6-mercaptopurine [6-MP] was added. Random assignment to the following groups, with follow up for 1.5 years: Chronic seton drainage for 1 year (15 cases); Anti-TNF therapy for 1 year (15 cases); Surgical closure after 2 months under a short course anti-TNF (14 cases). In the PISA prospective registry cohort, 20 patients chose chronic seton drainage, 21 anti-TNF treatment, and 9 surgical closure after anti-TNF induction.	The study was stopped by the data safety monitoring board because of futility.
			The primary outcome was the cumulative number of patients with fistula-related re-intervention[s] at 1.5 years.	
			In the randomized group, seton treatment was associated with the highest re-intervention rate [10/15, versus 6/15 anti-TNF and 3/14 surgical closure patients, p = 0.02]. No substantial differences in PDAI and quality of life between the three treatment groups were observed.	
			Interestingly, in the PISA prospective registry cohort, the re-intervention rate was similar between the groups (42% [8 cases], 48% [9 cases], and 44% [2 cases] in the chronic seton, anti−TNF, and the surgical closure after anti-TNF groups; p = 0.78). It is worth noticing that the inferiority of chronic seton treatment was not observed for any outcome measure.	
			Based on the results, the authors recommended that chronic seton treatment should not be recommended as the sole treatment for perianal Crohn’s fistulas.	

Biologicals

**Figure 3:**
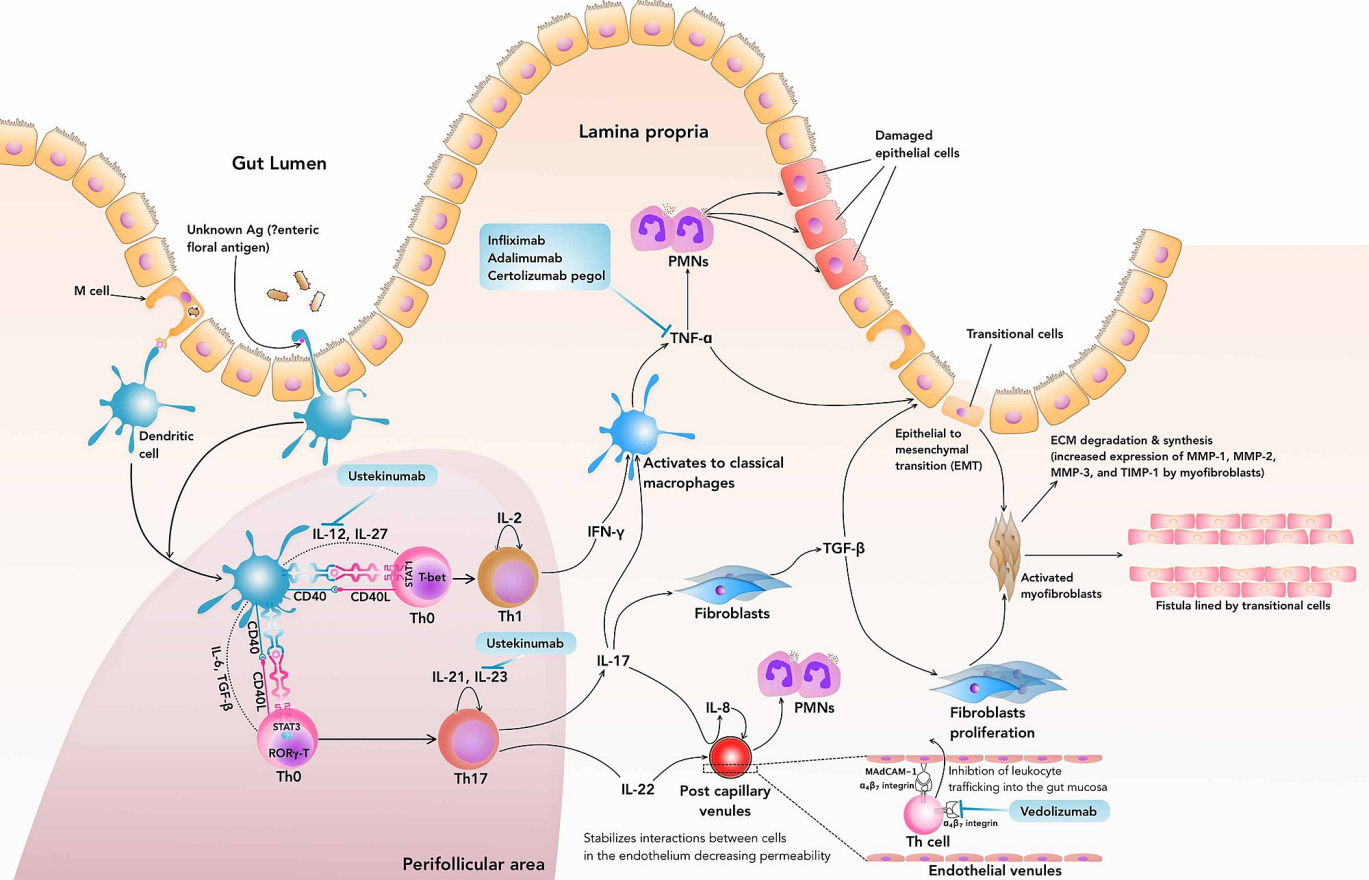
Immunopathogenesis of Crohn's Disease & Fistula Formation and the Development of Novel Target Immune therapies (Biologicals) affecting the Pathway Th: T Helper cells; IL: Interleukin; TNF: Tumor Necrosis Factor; IFN: Interferon; TGF: Transforming growth factor; CD: Cluster of Differentiation; Ag: Antigen; MMPs: Matrix Metalloproteinases; TIMPs: Tissue Inhibitors of Matrix Metalloproteinases; MAdCAM: Mucosal Addressin Cell Adhesion Molecule; PMNs: Polymorphonuclear leukocytes; STAT: Signal transducer and activator of transcription (a transcription factor); T-bet: (T-box expressed in T cells, a T-box transcription factor); RORγ: RAR-related orphan receptor gamma (a member of the nuclear receptor family of transcription factors).

The pathogenesis (Figure 3) of Crohn's disease involves Th1 and Th17 hypersensitivity due to an unknown antigen (possibly enteric floral antigens) within the intestinal mucosa. Increased production of TGF-β and IL-6 is responsible for the commitment of naive Th cells (Th0 cells) to Th17 cells, while IL-12 is required for differentiation of a Th0 cell into a Th1 cell. The production of IL-21 and IL-23 is responsible for the maintenance and expansion of the Th17 cells, while the local production of TNF-α mediates and propagates the inflammation [14]. In the inflammatory infiltrate, IL-12, TNF, and IL‑13 induce epithelial-to-mesenchymal transition and upregulation of matrix metalloproteinases, leading to tissue remodeling and fistula formation [15]. Therefore any pharmacological agent interrupting this pathway will theoretically resolve the inflammation and the progression of the disease. The antagonist to TNF-α has been approved for the management of Crohn's disease and its complications.

In a study conducted by Present et al. on patients with perianal Crohn’s fistula, infliximab IV at 5mg/kg resulted in a ≥ 50% reduction in the number of draining fistula in 68% of the patients as compared to the placebo (p=0.02), while 55% had the fistula closure [33]. In another study by Farrell et al., 70% (23/33) of patients with fistulous disease experienced >50% reduction in their Perianal Disease Activity Index (PDAI) at two weeks with 5mg/kg infusion of infliximab [34]. Similarly, in a prospective cohort study by Luna-Chadid et al., after the third infusion of infliximab (5mg/kg), the response was partial (≥ 50% from baseline) in 26% (27/105) and complete (full closure) in 57% (60/105), i.e., 82% (87/105) were responders with infliximab four weeks post-infusion of the last dose [35]. A randomized controlled trial (RCT) by Sands et al. found the response rate to be 43% (130/305) in perianal Crohn’s fistulous patients who received three infusions of Infliximab at 0, two, and six weeks and the median time for the loss of response after receiving three infusions of infliximab was 14 weeks [36]. To conclude that infliximab does have a statistically significant effect on the healing rate as compared to placebo for the perianal Crohn’s fistula, but it also requires a prolonged maintenance dose after induction therapy to maintain the remission. A study by Yarur et al. [37] also proves an incremental gain in fistula healing with higher infliximab levels. The area under the curve (AUC) for the association between fistula healing and infliximab levels was 0.82 (p < 0.0001), while the AUC for the association of infliximab levels and fistula closure was 0.69 (p = 0.014). The patients who did not show any improvement or lower infliximab levels in serum were having anti-infliximab antibodies and had a lower chance of achieving fistula healing (OR: 0.04 [95%CI: 0.005-0.3], p < 0.001).

Adalimumab (ADA) is a fully human anti-TNF IgG1 monoclonal antibody that has also been shown to induce and maintain clinical response in active CD not controlled by corticosteroids, immunosuppressants, or both, but the results of its overall effectiveness are questionable, while the effect on fistula improvement has not been proven so far. In an RCT (CLASSIC-I: Clinical assessment of Adalimumab Safety and efficacy Studied as Induction therapy in Crohn’s disease) conducted by the Hanauer et al. [38] on perianal Crohn's fistula patients, the rates of remission for the Crohn's disease at week four in the adalimumab 40mg/20mg, 80mg/40mg, and 160mg/80mg groups were 18% (p = 0.36), 24% (p = 0.06), and 36% (p = 0.001), respectively, and 12% in the placebo group. While the rates of fistula improvement at week four in the adalimumab 40mg/20mg, 80mg/40mg, and 160mg/80mg groups were 3/4 (75%), 2/10 (20%), and 1/12 (8%) respectively, and 2/6 (33%) in the placebo group, and the rates of fistula remission at week four in the adalimumab 40mg/20mg, 80mg/40mg, and 160mg/80mg groups were 3/4 (75%), 0/10 (0%), and 0/12 (0%) respectively, and 1/6 (17%) in the placebo group. Therefore adalimumab was not found to be superior to placebo in the management of the perianal fistula treatment. However, with a minimal beneficial effect on the rates of remission in moderate to severe Crohn's disease compared to placebo (note only the highest dose group at week four achieved statistical significance [p = 0.001] as compared to placebo). The CLASSIC-II trial (RCT) by Sandborn et al. [39] was a continuation of the CLASSIC-I trial to observe adalimumab's effect on the maintenance of remission. Group I consisted of 55 patients in remission were re-randomized and received adalimumab 40mg every other week (19 patients; Group 1a), 40mg weekly (18 patients; Group 1b), or placebo (18 patients; Group 1c) for 56 weeks, while the Group II consisted of the patients who were not in remission received open-label adalimumab 40mg at weeks 0 (week four of CLASSIC I) and two. There was a statistically significant difference in the remission rate in Group 1a (15/19, 79%), 1b (15/18, 83%) versus placebo (8/18, 44%) at week 56. Unfortunately, no direct data for fistula improvement or remission was presented in this study. In another RCT by Sandborn et al., [40] the rates of fistula improvement and remission at week four were 15% (3/20) of patients in the adalimumab group versus 20% (5/25) of patients in the placebo group for improvement and 5% (1/20) versus 8% (2/25), respectively, for remission. Although the sample was of small size, nevertheless, it explains that the use of the adalimumab for the improvement or remission of perianal Crohn's fistula is futile. In a large scale RCT (CHARM: Crohn’s Trial of the Fully Human Antibody Adalimumab for Remission Maintenance; phase III of CLASSIC-I trial) by Colombel et al. [41], complete fistula closure (closure of all fistulas that were draining at screening and baseline visits) were achieved in the randomized population at both week 26 and week 56 (30% [21/70] and 13% [6/47] for combined adalimumab groups and placebo group, respectively, at week 26 (p = 0.043) and 33% [23/70] and 13% [6/47] for combined adalimumab groups and placebo group, respectively, at week 56 (p = 0.016)). Of patients with complete fistula closure at week 26, 100% continued to have complete fistula closure at week 56 [41]. The major limitation of this study is that the many patients receiving placebo ceased treatment because of an adverse event (13.4%) than those receiving adalimumab, which might have increased the contrast between the treatment and placebo groups.

Certolizumab pegol is a pegylated humanized Fab' fragment that binds TNF-α. In an RCT conducted by Sandborn et al., 662 patients with moderate-severe Crohn's disease were assigned randomly to receive either 400mg of certolizumab pegol or placebo subcutaneously at weeks 0, two, and four and then every four weeks. In the overall population, at week six, induction response rates based on Crohn's Disease Activity Index (CDAI) were 35% in the certolizumab group and 27% in the placebo group (p = 0.02); at both weeks six and 26, the response rates were 23% and 16%, respectively (p = 0.02). Through week 26, 30% (14/46) of patients in the certolizumab group had fistula closure compared to 31% (19/61) of patients in the placebo group [42]. So as per the study, certolizumab does have a modest effect in response rates in patients with moderate to severe Crohn's disease, but the use of the certolizumab pegol for the fistula closure in the case of Crohn's does not show any effect in contrast to the placebo (statistically significant finding). The major limitation of the trial was that many patients were also receiving concurrent therapy, so the statistically significant effect of certolizumab therapy presented in the study can not be conclusively established whether it was the effect of the certolizumab or the concurrent therapy or the combination of the therapy. However, the concurrent treatment was proportionately divided into the placebo and the certolizumab group. Schreiber et al. [43] performed a similar RCT; 668 adults with moderate-severe Crohn's disease entered the induction phase. Fourteen percent (58/425; 28 in the certolizumab group and 30 in the placebo group) of patients who responded to induction therapy with certolizumab pegol had draining fistulas at baseline. In the certolizumab group, 54% (15/28) had closure of the fistula (defined as the absence of drainage on gentle compression at any two consecutive visits three weeks apart), compared with 43% (13/30) in the placebo group. Again no statistical significance was noted for the fistula closure with certolizumab pegol therapy as compared to the placebo. However, another subsequent RCT by the same author included 108 adults with draining Crohn's fistula, out of which nonresponders (50/108) at week six to open-label certolizumab therapy were excluded. The responders (the majority 55/58 had perianal fistula) were then randomized to certolizumab pegol 400mg (n = 28) or placebo (n = 30) every four weeks. It was found that at week 26, 36% of patients in the certolizumab pegol group had 100% fistula closure compared with 17% of patients receiving placebo (p = 0.038). The major limitations of the results are the exclusion of the nonresponders and the small sample size to confer any statistical significance. The study also concluded that the protocol-defined fistula closure (≥50% closure at two consecutive post-baseline visits ≥three weeks apart) was not statistically significant (p = 0.069), with 54% and 43% of patients treated with certolizumab pegol and placebo achieving this endpoint, respectively [44].

Vedolizumab is a monoclonal antibody directed against the α₄β₇ integrin on the T cells, inhibiting the T cells' transmigration, limiting the trafficking into the GI mucosa. In an RCT (NCT00783692 trial) conducted by Sandborn et al. [45], 368 patients (group 1) were randomly assigned to receive either IV vedolizumab (300mg; Group 1a; 220 cases) or placebo (Group 1b; 148 cases) at weeks 0 and two, while 747 patients (Group 2) received the open-label vedolizumab at weeks 0 and two. At week six, 461 showed a response to vedolizumab (vedolizumab responders), who were then randomly assigned to receive either IV vedolizumab (300mg) every four weeks (Group 2a; 154 cases) or every eight weeks (Group 2b; 154 cases) or placebo (Group 2c; 153 cases) until week 52. At the end of the induction phase, at six weeks, 14.5% (32/220) in Group 1a and 6.8% (10/148) in Group 1b had a clinical remission, i.e., CDAI of ≤150 (p = 0.02). While in Group 2, 17.7% (132/747) had a clinical remission. At the end of the maintenance phase, at week 52, 36.4% (56/154) in Group 2a and 39% (60/154) in Group 2b achieved clinical remission, as compared to 21.6% (33/153) in Group 2c (p < 0.001 and p = 0.004 for the comparison of the two vedolizumab groups, respectively, with placebo). While at week 52, the fistula closure rates in Group 2a and 2b were 22.7% (5/22) and 41.2% (7/17) compared to 11.1% (2/18) in Group 2c (p = 0.32 and p = 0.03, respectively). Although with a modest effect on systemic disease, vedolizumab-treated active Crohn's disease patients were more likely to be in remission than placebo during induction and maintenance. While vedolizumab's effect on fistula closure rate in the maintenance phase of vedolizumab responders did not reach statistical significance in Group 2a, but with a minimal statistically significant impact on fistula closure rate in Group 2b compared to placebo. Vedolizumab, compared to placebo, was associated with a higher rate of serious adverse events (24.4% vs. 15.3%), infections (44.1% vs. 40.2%), and serious infections (5.5% vs. 3.0%).

In a retrospective analysis of the NCT00783692 trial (2018) by Feagan et al. [46], at the end of the induction phase at week six, 12% (57/461) of vedolizumab responders (Group 2) had ≥ one active draining fistula, which was randomly assigned to receive either IV vedolizumab (300mg) every four weeks (22 cases) or every eight weeks (17 cases) or placebo (18 cases) until week 52. Note, 79% (45/57) had perianal draining Crohn's fistula, of which 32 were allotted in vedolizumab-treated group, while 13 were in the placebo group. By week 14, 28% (11/39) of vedolizumab-treated patients compared with 11% (2/18) of placebo-treated patients (absolute risk reduction [ARR] 95% confidence interval [CI], -11.4 to 43.9) achieved fistula closure. Respective rates at week 52 were 31% (12/39) and 11% (2/18) (ARR: 19.7%; 95% CI, -8.9 to 46.2). Similar results were observed in the patients with only perianal fistulae: 34% (11/32) in the vedolizumab-treated patients compared to 15% (2/13) in the placebo group. Respective values at week 52 were 31% (10/32) in the vedolizumab-treated while 11% (2/13) in the placebo group [ARR: 19.7%; 95% CI, -8.9 to 46.2]. There are several key points worth noting about this retrospective analysis; first, the response rate to vedolizumab in the NCT00783692 trial at the end of the induction phase was 47.67% (461/(220 + 747)) (CDAI ≤ 150). Only 45 had active perianal draining Crohn's fistula. With the vedolizumab responders, the fistula closure rate was significantly lower (31%) at the end of week 52 in the maintenance phase of the responders. The confidence interval of the absolute risk reduction at week 14 crosses 0, indicating not statistically significant, although the p-value of the ARR was not calculated. Albeit the sample's small size to confer a strong statistical relevance, the modest achievement in fistula closure and its rate was worthwhile. But if we include the vedolizumab non-responders in our calculation, the fistula closure rate at week 52 becomes 12/(39 + 85) = 9.67% (≈ 10%). It would decrease further if we include only Crohn's fistula. The question arises would it be worthwhile to supplement the patient of perianal Crohn's fistula with vedolizumab considering the low response rate (< 9.67%) and severe adverse reaction (24.4%).

To sum up, among biologicals, infliximab is the only therapy that has a statistically significant effect on the healing rate of perianal Crohn's fistula compared to placebo, but it also requires a prolonged maintenance dose after induction therapy to maintain the remission. The efficacy of adalimumab and certolizumab pegol for the healing or closure of perianal fistula closure has not been established in the study since the statistical significance is lacking compared to placebo. The efficacy of vedolizumab, albeit significantly lower but statistically significant for the remission of the perianal Crohn's fistula, is counterbalanced by the severe adverse reaction associated with its use. Therefore the use of either therapy for the sole purpose of fistula healing or closure in case of Crohn's disease would be futile.

Setons and Biologicals

The reported re-intervention rates in the case of perianal Crohn's fistula with seton drainage were 10-20% [28] compared with anti-TNF or surgical closure 30-50%. This is in contrast with an RCT (PISA trial) by Wasmann et al. where 44/126 patients with high perianal Crohn's fistulas were randomly assigned between 2013-2017 into three groups: chronic seton drainage for one year (15 assigned; Group 1a); anti-TNF therapy for one year (15 assigned; Group 1b); and surgical closure after two months under a short course anti-TNF (14 assigned; Group 1c). Fifty of 126 declined randomization due to treatment preference hence were included in a parallel prospective PISA registry cohort and chose one of the three options (20 patients [Group 2a], 21 patients [Group 2b], and nine patients [Group 2c]) [10].

The primary outcome measure was the fistula-related re-intervention rate at 1.5 years. It was determined that the seton treatment was associated with the highest re-intervention rate (10/15, versus 6/15 anti-TNF and 3/14 surgical closure patients, p = 0.02), while the re-intervention rates in the PISA cohort were 42%, 48%, and 44%. No substantial differences in PDAI and quality of life between the three treatment groups were observed. It was found that the seton treatment was associated with the highest re-intervention rate (10/15, versus 6/15 anti-TNF and 3/14 surgical closure patients, p = 0.02). It is worth mentioning that chronic seton treatment's inferiority was not observed for any outcome measure in the PISA cohort [10]. The limitation with this study is that the study included a small sample size in the randomized group (44 cases), hence the low power to confer strong statistical relevance, seven patients in Group 1a also received the anti-TNF therapy, four patients in Group 1b also received the additional seton. The sample size needed to detect the statistically significant difference in re-intervention rate with two-sided chi-squared testing equals 42 patients per group, or 126 patients overall (alpha 0.05, power 80% and 5% dropout rate), while the patients in each group did not full-fill the criteria [47] (as suggested by the authors in their first publication). This intermingling of the therapy within the group on a small sample blurred the picture of the re-intervention rate, hence unable to draw firm conclusions (Table 5).

**Table 5:** Clinical Studies on the Management of Perianal Crohn's Fistula Primarily by Biologicals or the Combination of Therapies ¥ Published date; CD (Crohn’s Disease); non-CD (Non-Crohn’s Disease); SQ (subcutaneous) , Crohn's Disease Activity Index (CDAI), PDAI (Perianal Disease Activity Index); CRP (C-reactive protein); CLASSIC: Clinical assessment of Adalimumab Safety and efficacy Studied as Induction therapy in Crohn’s disease; CHARM: Crohn’s Trial of the Fully Human Antibody Adalimumab for Remission Maintenance; RCT (randomized controlled trial)

Studies	Method	Patient Characteristics	Key Findings with Complications (if any)	Limitations
Present, et al. [33]	RCT (1999)	94 adult patients with draining perianal Crohn’s fistula of at least three months' duration	All patients were randomly assigned to receive IV infusions at weeks 0, 2, and 6 to one of the three groups: Group 1 (placebo; 31 patients), Group 2 (5 mg/kg of Infliximab; 31 patients), and Group 3 (10 mg/kg of Infliximab; 32 patients)	
			The primary endpoint was a reduction of ≥ 50% from baseline in the number of draining fistulas observed at ≥ 2 consecutive study visits. The secondary endpoint was the closure of all fistulas.	
			68% (21/31) of Group 2 and 56% (18/32) of Group 3 achieved the primary endpoint, as compared with 26% (8/31) of Group 1 (P=0.002 and P=0.02, respectively). While 55% (17/31) of Group 2 and 38% (12/32) of Group 3 had closure of all fistulas, as compared with 13% (4/31) of Group 1 (P=0.001 and P=0.04, respectively). The median length of time during which the fistulas remained closed was three months.	
			> 60% of patients in all the groups had adverse events, most commonly headache, abscess, upper respiratory tract infection, and fatigue, especially in patients treated with Infliximab.	
Farrell et al. [34]	Case series (2000)	100 patients with CD (53 women and 47 men; mean age, 41 year)	All patients were divided into three groups, namely active disease (57 patients), perianal fistulous disease (33 patients), and steroid dependency (10 patients) group, based on the clinical status and received IV infusions (5 mg/kg) at weeks 0, 2, and 6, with a total of 233 infusions.	
			All patients in the perianal fistulous disease group had 50 draining fistulas (19 had one fistula, 11 had two fistulas, and 3 had three fistulas).	
			70% (23/33) cases in the fistulous disease group experienced >50% reduction in their PDAI at two weeks; the mean duration of response was 10.9 weeks. 78% (18/23) of this group maintained this reduction at 18 weeks.	
			6.9% of infusions (16/233) resulted in adverse reactions, including 14 experienced infectious adverse events, 13 of whom were on concurrent steroids, 1 experienced an anaphylactic shock.	
Sands et al. [36]	RCT (2004)	306 adult patients with draining perianal Crohn’s fistula of at least three months' duration	After receiving 5 mg of infliximab/kg IV (induction) on weeks 0, 2, and 6, all patients were randomly assigned based on the response status at week 14 into two groups (responders [195 patients] and non-responders [87 patients]) to receive either a placebo or 5 mg of Infliximab/kg (maintenance) every 8 weeks and to be followed to 54 weeks.	
			Responders demonstrated rapid onset of response with an increase in response rate after every infusion (induction).	
			Before randomization, a complete response was observed in 31% (95/306), 43% (130/305; 1 patient discontinued treatment), and 48% (147/305) at weeks 2, 6, and 14, respectively.	
			After randomization, the median time to the loss of response, hence a need for a change in the treatment of CD (the primary analysis) was 14 weeks in the placebo maintenance group, compared to > 40 weeks in the Infliximab maintenance group (p < 0.001) after randomization.	
Luna-Chadid et al. [35]	Prospective cohort (2004)	108 adult patients with fistulizing Crohn’s disease (18% inflammatory, rest with no signs of inflammation)	All patients received 5 mg of infliximab/kg IV (induction) on weeks 0, 2, and 6. Partial response was defined as a reduction of ≥ 50% from the base-line in the number of draining fistulae, while a complete response was defined as the closure of all fistulae.	The percentages of patients receiving concurrent therapy with other drugs were: azathioprine/6-mercaptopurine (68%), corticosteroids (55%), 5-aminosalicylates (75%), metronidazole (67%), and ciprofloxacin (32%).
			26% (27/105) and 57% (60/105) demonstrated a partial and complete response (total 82% responders), respectively, four weeks post-infusion of the last dose of Infliximab.	
			Based on the fistula location, the response rates (partial/complete%) were: enterocutaneous (25/68%), perianal (35/60%), rectovaginal (36/64%), and enterovesical (20/40%).	
			In the multivariate analysis, none of the studied variables (including concomitant immunosuppressive therapy) correlated with Infliximab's efficacy.	
			The incidence of adverse effects (21%) depending on the dose of Infliximab was: first dose (5.6%), second (7.4%), and third (11.1%).	
Yarur et al. [37]	Cross-sectional (2017)	117 adult patients with perianal Crohn’s fistula	Managed with inﬂiximab for at least 24 weeks.	
			Significantly higher median serum infliximab levels were found in patients exhibiting healing of the fistula compared to those with active fistulas [15.8 vs. 4.4 μg/mL, respectively (P < 0.0001)].	
			The Infliximab levels were directly correlated with the fistula healing rate. The area under the curve (AUC) for the association between fistula healing and infliximab levels was 0.82 (P < 0.0001), while the AUC for the association of infliximab levels and fistula closure was 0.69 (P = 0.014). Achieving inﬂiximab levels ≥ 10.1 µg/mL in patients with CD and perianal ﬁstulas may improve outcomes as part of a treat-to-target strategy.	
			Patients with anti-infliximab antibodies had a lower chance of achieving fistula healing (OR: 0.04 [95%CI: 0.005-0.3], P < 0.001).	
Hanauer et al. [38]	RCT (2006): CLASSIC-I trial	299 patients with moderate to severe CD naive to anti-TNF therapy, with 11% (32/299) had draining enterocutaneous or perianal fistulas and were unevenly distributed across the treatment groups.	All patients were randomized to receive SQ injections of Adalimumab 40 → 20 mg, 80 → 40 mg, or 160 → 80 mg or placebo → placebo at weeks 0 and 2, respectively.	Disproportionate distribution of patients among groups.
			The primary endpoint was the determination of a significant difference in remission rates (defined as a CDAI score <150 points) at week 4 among the four groups. At weeks 4, the rates of remission of the CD in the adalimumab 40 → 20 mg, 80 → 40 mg, and 160 → 80 mg groups were 18% (P = .36), 24% (P = .06), and 36% (P = .001), respectively, while 12% in the placebo group. All four groups experienced adverse effects at similar frequencies except injection site reactions, which were more common in adalimumab-treated patients.	
			At week 4, the rates of fistula improvement in the adalimumab 40 → 20 mg, 80 → 40 mg, and 160 → 80 mg groups were 75% (3/4), 20% (2/10), and 8% (1/12) respectively, and 33% (2/6) in the placebo group. While the rates of fistula remission at weeks 4 in the adalimumab 40 → 20 mg, 80 → 40 mg, and 160 → 80 mg groups were 7%% (3/4), 0% (0/10), and 0% (0/12) respectively, and 17% (1/6) in the placebo group. Therefore, the rates of fistula improvement and remission for the adalimumab-treated patients and those receiving placebo were not significantly different.	
			Adalimumab was not found to be superior to placebo in the management of the perianal fistula treatment..	
Sandborn et al. [39]	RCT (2007): CLASSIC-II trial (phase 2 of CLASSIC-I)	276 patients from CLASSIC-I trial	All received open-label Adalimumab 40 mg at weeks 0 (week 4 of CLASSIC I trial) and 2	No control group in the group 2. No direct data for fistula improvement or remission was presented in the study.
			55 patients in remission (Group 1) at both weeks 0 and 4 were re-randomized to adalimumab 40 mg every other week (19 patients; Group 1a), 40 mg weekly (18 patients; Group 1b), or placebo (18 patients; Group 1c) for 56 weeks. The primary endpoint was the maintenance of remission (CDAI <150) in randomized patients through week 56.	
			204 patients (Group 2) who were not in remission were ineligible for randomization hence starts receiving at week 4, open‐label adalimumab 40 mg every other week.	
			The perianal fistula was present in only 9% (5/55; 3 in group 1c and 2 in group 1a) in group 1 while 15% (30/204) in group 2.	
			In group 1, at week 56, there was a significant difference in the remission rates between the Group 1a (79% [15/19]), Group 1b (83% [15/18]), and the Group 1c (placebo; 44% [8/18]) (p<0.05 for each adalimumab group v placebo).	
			In group 2, 64% (131/204) completed 56 weeks of treatment, 71 remained on their initial regimens, while 60 had their dosages increased to 40 mg weekly before week 56. 46% (93/204) were in remission at week 56.	
			In patients with moderate to severe Crohn's disease naive to anti-TNF treatment, Adalimumab induced and maintained clinical remission for up to 56 weeks.	
Sandborn et al. [40]	RCT (2007)	325 patients with moderate to severe CD	All patients were randomized to receive SQ injections of Adalimumab 160 → 80 mg (Group 1; 159 cases) or placebo → placebo (Group 2; 166 cases) at weeks 0 and 2, respectively, and followed patients through week 4.	
			The perianal fistula was present in 13% (20/159) in group 1, while 15% (25/166) in group 2. The previous loss of response to infliximab was present in 48% (77/159) in group 1, while 52% (87/166) in group 2.	
			At week 4, 21% (34/159) of patients in Group 1 compared to 7% (12/166) of patients in Group 2 achieved remission (P<0.001).	
			At week 4, rates of fistula improvement and remission were similar for both groups: 15% (3/20) in Group 1 and 20% (5/25) in Group 2 for improvement while 5% (1/20) in Group 1 and 8% (2/25) in Group 2 for remission.	
			Adalimumab was not found to be superior to placebo in the management of the perianal fistula treatment.	
Colombel et al. [41]	RCT (2007): CHARM trial ((phase 3 of CLASSIC-I))	854 patients with moderate to severe CD	All received open-label induction therapy with Adalimumab 80 mg (week 0) followed by 40 mg (week 2).	
			At week 4, 58% (499/854) responded to Adalimumab induction (responders), 33% (279/854) did not respond (nonresponders), while the remaining 76 excluded from the study.	
			At week 4, 778 patients were stratified by the response (decrease in CDAI >70 points from baseline) and randomized to double-blind treatment with adalimumab 40 mg every other week (Group 1; 260), Adalimumab 40 mg weekly (Group 2; 257) or placebo (Group 3; 261), through week 56.	
			The perianal fistula was present in 130 patients (15.4%) out of the total 854, 64 patients (12.8%) out of 499 week-4 randomized responders, and 53 patients (19%) out of 279 week-4 nonresponders.	
			At weeks 26 and 56, the percentage of week-4 randomized responders in remission (CDAI score 150), i.e., the primary endpoint was significantly greater in both adalimumab treatment groups versus placebo (Group 1 [40%], Group 2 [47%], and Group 3 [17%] at week 26; Group 1 [36%], Group 2 [41%], and Group 3 [12%] at week 56; P<0.001 for pairwise comparison between each Adalimumab treatment group and placebo).	
			At both weeks 26 and 56, a larger number of Adalimumab-treated patients (Group 1 & 2) achieved complete fistula closure (closure of all fistulas that were draining at screening and baseline visits) compared to Group 3 (received placebo) in the randomized population (30% [21/70] and 13% [6/47] for combined Adalimumab groups and placebo group, respectively, at week 26 (P=0.043) and 33% [23/70] and 13% [6/47] for combined adalimumab groups and placebo group, respectively, at week 56 (P=0.016)). Of patients with complete fistula closure at week 26, 100% continued to have complete fistula closure at week 56.	
Fortea-Ormaechea et al. [48]	Retrospective cohort (2011)	174 adult patients with Crohn’s disease. 87 (50%) had developed perianal fistulizing disease, and among them 53 (30.5%) had active draining fistulas. 59% (102) of patients received Infliximab therapy in the past, of which 43 (35%) discontunued due to loss of response.	All patients received 160 mg → 80 mg of Adalimumab (induction therapy) at weeks 0 and 2, respectively.	
			The maintenance therapy included 40 mg of Adalimumab every 2 weeks or weekly, depending on the increment in the dose required in case of loss of response to Adalimumab during follow up. So, the dose was escalated in 32.8% (57/174) of patients, and the median time to dose increment was 33 weeks (range 2-120).	
			The authors distinguished the luminal versus perianal fistulizing disease in evaluating the effectiveness of ADA. The complete response rate at 1 month, 6 months, and at the end of follow-up were 63.4%, 70.4%, and 63.3%, respectively, in luminal disease, while in the perianal fistulizing disease, these were 49%, 50%, and 41.5%, respectively.	
			No significant difference was noted by the authors in the effectiveness between those who received Adalimumab as first-line treatment and those who had previously received infliximab (50 and 56.3% of complete response at the end of follow up in luminal disease, p=0.829; and 64 and 33.3% in perianal fistulizing disease, p=0.164).	
Lichtiger et al. [49]	RCT (2010)	673 patients with moderate to severe Crohn’s disease	After an ≥ 8-week infliximab washout, all patients with moderate-to-severe CD received open-label adalimumab as induction (160 → 80 mg at weeks 0 and 2 respectively) and maintenance (40 mg every other week) therapies. After 8 weeks of treatment, patients with flare-ups or nonresponders could receive weekly treatment.	
			17% were infliximab primary nonresponders, and 83% were initial responders. 3% of patients had severe infections (mainly abscesses).	
			Complete fistula healing was achieved in 39% (34/88) patients with baseline fistulas. Improvements in quality of life and work productivity were sustained from week 4 to week 24 for all patients, as well as the subgroup of primary nonresponders.	
Sandborn et al. [42]	RCT (2007)	662 adults with moderate-to-severe Crohn's disease	After stratification according to CRP levels, all patients were randomly assigned to receive either 400 mg of certolizumab pegol or placebo SQ at weeks 0, 2, and 4 and then every 4 weeks.	
			The primary endpoints were the induction of response at week 6 and response at weeks 6 and 26.	
			Among patients with a baseline CRP level of at least 10 mg/L, 37% of patients in the certolizumab group responded at week 6 compared to 26% in the placebo group (P=0.04). At both weeks 6 and 26, the corresponding values were 22% and 12%, respectively (P=0.05). While in the overall population, response rates at week 6 were 35% in the certolizumab group and 27% in the placebo group (P=0.02); at both weeks 6 and 26, the response rates were 23% and 16%, respectively (P=0.02).	
			At weeks 6 and 26, remission rates in the two groups did not differ significantly (P=0.17).	
			Through week 26, 30% (14/46) of patients in the certolizumab group had fistula closure compared to 31% (19/61) of patients in the placebo group.	
			10% of patients in the Certolizumab group and 7% in the placebo group reported severe adverse events, while serious infections were noticed in 2% and < 1%, respectively. Antibodies to the drug and antinuclear antibodies developed in 8% and 2% of the patients in the Certolizumab group, respectively.	
Schreiber et al. [43]	RCT (2007)	668 adults with moderate-to-severe Crohn's disease entered the induction phase	All patients received 400 mg of Certolizumab pegol (induction therapy) SQ at weeks 0, 2, and 4, respectively.	
			At week 6, 64% (428/668) responded to Certolizumab pegol induction (responders), which were randomly assigned (after stratification according to CRP levels) to receive either 400 mg of certolizumab pegol (215/428) or placebo (210/428) every 4 weeks through week 24, with follow-up at week 26.	
			Among responders at week 6, the response was maintained through week 26 in 62% cases with a baseline CRP level of ≥ 10 mg/liter (the primary endpoint) who were receiving certolizumab pegol, compared to 34% of those receiving placebo (P<0.001).	
			Among responders at week 6, the remission (CDAI ≤ 150) at week 26 was achieved in 48% of patients who were receiving certolizumab pegol compared to 29% of those receiving placebo (P<0.001).	
			14% (58/428) of responders at week 6 had draining fistulas at baseline (28 cases in the certolizumab group, 30 cases in the placebo group). During follow up, of the 58 patients, 54% (15/28) had closure of the fistula (absence of drainage on gentle compression at any two consecutive post-baseline visits at least 3 weeks apart) in the certolizumab group compared to 43% (13/30) in the placebo group. Note the statistical significance of this finding is not mentioned in the study.	
Schreiber et al. [44]	RCT (2011)	108 adults with draining Crohn's fistula	All patients received 400 mg of Certolizumab pegol (open-label induction therapy) SQ at weeks 0, 2, and 4. The response was defined as a ≥100-point decrease from baseline in the CDAI.	Exclusion of the nonresponders and small sample size to confer any statistical significance
			At week 6, nonresponders (50/108) were excluded. While responders with draining fistulas (58/108) were randomized to certolizumab pegol 400 mg (28/58) or placebo (30/58) every 4 weeks across weeks 8-24. Assessment of fistula closure was performed throughout the study, with a final evaluation at week 26.	
			Among responders, 95% (55/58) had perianal fistulas. At week 26, 36% (10/28) of patients in the certolizumab pegol group had 100% fistula closure than 17% (5/30) of patients who received placebo (P = 0.038).	
			≥50% closure at two consecutive post-baseline visits ≥ 3 weeks apart defines the Protocol-defined fistula closure, which was not statistically significant (P = 0.069), with 54% and 43% of patients treated with certolizumab pegol and placebo achieving this endpoint, respectively.	
			Continuous treatment with certolizumab pegol improves the likelihood of sustained perianal fistula closure compared with a placebo.	
Sandborn et al. [45]	RCT (2013): NCT00783692 trial	1115 adult patients enrolled with active Crohn’s disease, with 410 patients had a history of fistulizing disease, and 165 had active draining fistula.	In the induction phase, 368 patients (group 1) were randomly assigned to receive either IV Vedolizumab (300 mg; Group 1a; 220 cases) or placebo (Group 1b; 148 cases) at weeks 0 and 2, while 747 patients (group 2) received the open-label Vedolizumab at weeks 0 and 2. In the maintenance phase, 41.34% (461/1115; Group 2) who had a response at week 6 (Vedolizumab-responders) were then randomly assigned to receive either IV Vedolizumab (300 mg) every 4 weeks (Group 2a; 154 cases) or every 8 weeks (Group 2b; 154 cases) or placebo (Group 2c; 153 cases) until week 52.	
			At the end of the induction phase, at 6 weeks, 14.5% (32/220) in Group 1a and 6.8% (10/148) in Group 1b had a clinical remission, i.e., CDAI of ≤150 (P=0.02). While in group 2, 17.7% (132/747) had a clinical remission with a CDAI-100 in 34.4% (257/747).	
			At the end of the maintenance phase, at week 52, 36.4% (56/154) in Group 2a and 39% (60/154) in Group 2b achieved clinical remission, as compared to 21.6% (33/153) in Group 2c (P<0.001 and P=0.004 for the comparison of the two vedolizumab groups, respectively, with placebo).	
			At week 52, the fistula closure rate in Group 2a and 2b was 22.7% (5/22) and 41.2% (7/17) compared to 11.1% (2/18) in Group 2c (P=0.32 and P=0.03 respectively).	
			Compared to placebo, Vedolizumab was associated with a higher rate of serious adverse events (24.4% vs. 15.3%), infections (44.1% vs. 40.2%), and serious infections (5.5% vs. 3.0%).	
Feagan et al [46]	Retrospective exploratory analysis of the NCT00783692 trial (2018)	Vedolizumab responders (461/1115) of the NCT00783692 trial	At the beginning of the maintenance phase, 12% (57/461) of Vedolizumab responders (Group 2 of NCT00783692 trial) had ≥ 1 active draining fistula, which was randomly assigned to receive either IV Vedolizumab (300 mg) every 4 weeks (22 cases) or every 8 weeks (17 cases) or placebo (18 cases) until week 52. Note 79% (45/57) had perianal draining Crohn’s fistula. Of which, 32 were allotted in Vedolizumab treated group, while 13 were in the placebo group.	
			By Week 14, 28% (11/39) of Vedolizumab-treated patients compared with 11% (2/18) of placebo treated patients (absolute risk reduction [ARR] 95% confidence interval [CI], –11.4 to 43.9) achieved fistula closure. Corresponding rates at Week 52 were 31% (12/39) and 11% (2/18) (ARR: 19.7%; 95% CI, –8.9 to 46.2).	
			Similar results were observed in the patients with only perianal fistulae, 34% (11/32) in the Vedolizumab-treated patients compared to 15% (2/13) in the placebo group. Corresponding values at Week 52 were 31% (10/32) in the Vedolizumab treated while 11% (2/13) in the placebo group [ARR: 19.7%; 95% CI, –8.9 to 46.2].	

## Conclusions

To conclude, chronic seton therapy should be the primary approach, especially if the patient has a perianal abscess, supported by the low incidence of reintervention, recurrent abscess formation, side-branching of the fistulous tract, preservation of the fistulous tract's patency, and cost-effectiveness. The significant disadvantages of seton therapy are the discomfort and the time it takes to achieve stability.

Among the biologicals, infliximab is the only therapy which has a statistically significant effect on the healing rate of perianal Crohn's fistula compared to placebo, but the major disadvantage associated with anti-TNF as sole therapy is high re-intervention rate, prolong maintenance therapy, high recurrence rate, and severe side effects. The efficacy of adalimumab and certolizumab pegol for the healing or closure of the perianal fistula closure has not been established in the clinical trials since the statistical significance is lacking compared to placebo. The efficacy of vedolizumab, albeit significantly lower but statistically significant for the remission of the perianal Crohn's fistula, is counterbalanced by the severe adverse reaction associated with its use. Therefore the use of either therapy for the sole purpose of fistula healing or closure in case of Crohn's disease would be futile.

We hypothesize that the two aspects should be addressed concomitantly to increase the rate of fistula closure. First, the seton should be used as initial therapy to maintain the tract's patency to allow the abscess's drainage and minimize the intestinal flora colonization within the tract mucosa, thereby leukocytic infiltration and propagation of inflammation within the tract. The absorbable seton could be used instead of non-absorbable. The second aspect that has to be considered is that we should target the initial stimulation of the Th1/Th17 mediated hypersensitivity instead of a factor/cytokine involved in the mediation of the inflammation. Although the unknown antigen triggering such hypersensitivity is not clear, we could target the RORγ-T (transcription factor involved in activation of Th17 cells) and the T-bet (transcription factor involved in activation of Th17 cells) within the GI mucosa by a novel target immune therapy.
